# Diagnosing norovirus-associated infectious intestinal disease using viral load

**DOI:** 10.1186/1471-2334-9-63

**Published:** 2009-05-14

**Authors:** Gemma Phillips, Ben Lopman, Clarence C Tam, Miren Iturriza-Gomara, David Brown, Jim Gray

**Affiliations:** 1 Department of Gastrointestinal, Emerging and Zoonotic Infections, Health Protection Agency Centre for Infections, 61 Colindale Avenue, London, UK; 2 Infectious Disease Epidemiology Unit, London School of Hygiene and Tropical Medicine, Keppel Street, London, UK; 3 Virus Reference Department, Health Protection Agency Centre for Infections, 61 Colindale Avenue, London, UK

## Abstract

**Background:**

Reverse transcription-polymerase chain reaction (RT-PCR) is the main method for laboratory diagnosis of norovirus-associated infectious intestinal disease (IID). However, up to 16% of healthy individuals in the community, with no recent history of IID, may be RT-PCR positive; so it is unclear whether norovirus is actually the cause of illness in an IID case when they are RT-PCR positive. It is important to identify the pathogen causing illness in sporadic IID cases, for clinical management and for community based incidence studies. The aim of this study was to investigate how faecal viral load can be used to determine when norovirus is the most likely cause of illness in an IID case.

**Methods:**

Real-time RT-PCR was used to determine the viral load in faecal specimens collected from 589 IID cases and 159 healthy controls, who were infected with genogroup II noroviruses. Cycle threshold (Ct) values from the real-time RT-PCR were used as a proxy measure of viral load. Receiver-operating characteristic (ROC) analysis was used to identify a cut-off in viral load for attributing illness to norovirus in IID cases.

**Results:**

One hundred and sixty-nine IID cases and 159 controls met the inclusion criteria for the ROC analysis. The optimal Ct value cut-off for attributing IID to norovirus was 31. The same cut-off was selected when using healthy controls, or IID cases who were positive by culture for bacterial pathogens, as the reference negative group. This alternative reference negative group can be identified amongst specimens routinely received in clinical virology laboratories.

**Conclusion:**

We demonstrated that ROC analysis can be used to select a cut-off for a norovirus real time RT-PCR assay, to aid clinical interpretation and diagnose when norovirus is the cause of IID. Specimens routinely received for diagnosis in clinical virology laboratories can be used to select an appropriate cut-off. Individual laboratories can use this method to define in-house cut-offs for their assays, to provide the best possible diagnostic service to clinicians and public health workers. Other clinical and epidemiological information should also be considered for patients with Ct values close to the cut-off, for the most accurate diagnosis of IID aetiology.

## Background

Infectious intestinal disease (IID) is a syndrome of mixed aetiology; many different pathogens can infect the human gastrointestinal tract and produce diarrhoea, vomiting and other characteristic symptoms. Mixed gastrointestinal infections are frequently detected, especially in infants and young children and when polymerase chain reaction (PCR) assays are used for diagnosis [1,2]. It is important to determine which pathogen is the cause of illness, in order to direct clinical management for individual patients and to advance epidemiological understanding of IID.

Reverse transcription- PCR (RT-PCR) is now the method of choice for detecting norovirus in clinical specimens. RT-PCR detects norovirus at lower concentrations and is less affected by specimen quality and preparation than electron microscopy [3-5]; large numbers of specimens can be tested simultaneously, compared to the single throughput for electron microscopy. RT-PCR also detects a much wider range of norovirus genetic variants than enzyme-linked immunosorbent assays (ELISA) and may be more easily adaptable for detection of new strains [6].

However, many healthy individuals, with no recent history of IID, are RT-PCR positive [7-9], meaning that virus detection by RT-PCR is not well correlated with disease in norovirus infection. If RT-PCR positivity does not necessarily equate to norovirus-associated IID, it cannot be used alone to attribute illness to norovirus in IID cases; it is possible that the norovirus infection is 'asymptomatic' in the IID case, with another pathogen, detected or undetected, actually causing the symptoms. The poor diagnostic specificity of PCR and the associated difficulties for clinical interpretation of test results have been highlighted for other viral pathogens [10,11].

Previous studies have demonstrated differences in faecal norovirus load between symptomatically and asymptomatically infected individuals [7,12]. Histopathological investigations of experimentally inoculated volunteers and naturally infected individuals also indicate that the mechanism of pathogenesis in norovirus infection may rely on damage to the intestinal epithelium, caused by viral replication [13-15], so that symptoms may be a result of high viral loads. The aim of this study was to use faecal viral load measurements to determine when illness is attributable to norovirus in IID cases.

## Methods

### Specimens

Faecal specimens were collected from IID cases and healthy controls during the Infectious Intestinal Disease Study for England (1993–1996) [16]. IID cases were recruited from a prospectively followed cohort in the community, or on consultation with their general practitioner for IID. IID cases had acute diarrhoea or vomiting, lasting less than two weeks, with no known non-infectious cause, preceded by a symptom free period of at least three weeks [17]. Healthy controls, with no history of IID for the preceding three weeks, were recruited from within the community cohort or from the registration lists of participating general practices (but not after consultation for another condition) [17]. Controls were recruited concurrently to IID cases. IID cases were asked to provide a faecal specimen during acute illness and controls provided a specimen at recruitment.

### Testing

In the original study, norovirus was detected using electron microscopy. Faecal specimens were also tested for a range of other bacterial, viral and protozoal pathogens, using bacterial culture, microscopy or ELISA. Specimens with sufficient volume remaining after testing were archived in frozen storage [18]. Subsequently the archived specimens were all re-tested for norovirus using RT-PCR [7,19]. PCR testing was also used to detect seven other common bacterial, viral and protozoal pathogens.

For the present study, norovirus RNA was re-extracted from the stored faecal specimens that were previously positive for norovirus by EM or RT-PCR, and real-time RT-PCR (method previously described [7]) was used to determine the viral load. The real time RT-PCR assay has separate primer pairs for norovirus genogroup I and genogroup II, so it was possible to use the assay to identify the genogroup of norovirus present. Only viral load measurements from norovirus genogroup II positive specimens were used for this analysis; differences in the performance of the two genogroup specific assays mean that it is not appropriate to directly compare the results between the two genogroups (J. Gray, personal communication). Specimen collection and testing for norovirus is summarised in Additional File 1.

### Data

The cycle threshold (Ct) value from the real time RT-PCR was used as a proxy measure of faecal viral load. The Ct value is inversely proportional to the amount of virus present in the specimen, so the lower the Ct value the higher the faecal viral load. The Ct value represents the number of rounds of PCR replication required to raise the number of copies of the target sequence in the reaction mixture above a pre-determined threshold [20]. The real time RT-PCR assay was run for 40 cycles, so the maximum possible Ct value for positive specimens in this study was 39.

### Descriptive analysis

The median Ct value and inter-quartile range were calculated for IID cases and controls; comparisons were made between groups using the rank-sum test in Stata 10 [21].

### Receiver-operating characteristic analysis

Receiver-operating characteristic (ROC) analysis was used to define a cut-off in the Ct values, to attribute disease to norovirus in IID cases. There is no gold standard test for diagnosing norovirus-associated IID. We therefore used microbiological and clinical characteristics to select reference groups for the ROC analysis.

#### Reference positive groups

We defined three reference positive groups, selected to have Ct values that are representative of where norovirus is causing illness (Table 1). Reference positive group 1 included only IID cases who were diagnosed as norovirus positive by electron microscopy; the high viral loads required for detection by electron microscopy correspond to viral shedding during acute infection in experimentally inoculated volunteers [22,23], so these IID cases are highly likely to have IID caused by norovirus.

**Table 1:** Inclusion criteria for the ROC analysis reference groups

Reference group	Inclusion Criteria
Reference positive 1	1. IID 2. Norovirus detected by electron microscopy 3. Norovirus infection confirmed by RT-PCR
	
Reference positive 2	1. IID 2. Norovirus detected by electron microscopy 3. Norovirus infection confirmed by RT-PCR Or 1. IID 2. Electron microscopy negative 3. Norovirus detected by RT-PCR 4. No other pathogen detected 5. Specimen collected within 3 days of symptom onset
	
Reference positive 3	1. IID 2. Norovirus detected by electron microscopy and/or RT-PCR 3. Negative for *Campylobacter *spp., *Salmonella *spp. and *Shigella *spp. by bacterial culture and *Cryptosporidium *spp. by light microscopy (and rotavirus A by ELISA in children aged less than five years only)
	
Reference negative 1	1. No history of IID in previous 3 weeks 2. Norovirus detected by RT-PCR
	
Reference negative 2	1. IID 2. Norovirus detected by RT-PCR 3. Infection with *Salmonella *spp., *Campylobacter *spp. or *Shigella *spp. detected by bacterial culture or *Cryptosporidium *spp. detected by light microscopy (or rotavirus A by ELISA in children aged less than five years only)

In reference positive group 2, we additionally included IID cases who were electron microscopy negative and subsequently RT-PCR positive, providing that they had no other pathogens identified in their stool and that they had collected a specimen early in their illness (less than three days since symptom onset). These two restrictions were used to ensure that norovirus was the most likely cause of their illness and to ensure that their faecal viral load is representative of acute symptomatic norovirus infection [12,22,23]. We defined this second reference group to determine whether using only electron microscopy positive cases in reference group 1 biased the cut-off to lower Ct values (higher viral loads).

Reference positive group 3 included IID cases who were RT-PCR positive for norovirus (including those previously positive by EM) and who were negative for other bacterial, protozoal and viral pathogens that are routinely detected in clinical diagnostic algorithms for sporadic IID in National Health Service laboratories in the UK [24,25]. This restriction was used to make norovirus the most likely cause of illness in these IID cases, so that their Ct values should be representative of where norovirus is causing illness. We defined this third reference positive group to explore whether it is suitable for selecting a Ct value cut-off, because electron microscopy diagnosis is no longer used in clinical laboratories in the UK, so cannot be used to select a reference positive group in future studies.

#### Reference negative groups

We defined two reference negative groups, selected to have Ct values representative of where norovirus is not causing illness (Table 1). Reference negative group 1 included norovirus-infected healthy controls. Reference negative group 2 included norovirus infected IID cases with a bacterial infection diagnosed by culture or rotavirus A infection diagnosed by ELISA (for children aged less than five years only). Bacterial culture without enrichment may indicate the presence of high concentrations of viable bacterial cells, meaning that the bacteria detected are likely to be causing illness, rather than the norovirus infection. Similarly, ELISA for rotavirus A has a high detection limit that correlates well with disease [26,27], so rotavirus A is probably the cause of illness in ELISA positive individuals, rather than the norovirus infection. We defined this second reference negative group to explore whether it is suitable for selecting a cut-off, because specimens from healthy controls are not routinely received in clinical laboratories, so cannot be used as the reference negative group if other laboratories want to use this method to develop a cut-off for their real time assays.

In each ROC analysis, the sensitivity and specificity were calculated for each potential cut-off in the range of Ct values and an empirical ROC plot created using Stata 10 [21]. The Youden index (sensitivity + specificity-1) was calculated and the maximum value used to identify the optimal cut-off [28-30]. The analysis was done for all ages together and then separately, in children aged less than five years and individuals aged five years or older.

### Ethics

Ethical approval was granted from both local and national research ethics committees (Royal College of General Practitioners, London School of Hygiene and Tropical Medicine, Public Health Laboratory Service) for the IID study, including creation of the faecal specimen archive [17]. Written, informed consent was obtained from all cases and controls. The faecal specimen archive was anonymised and no further ethical approval was sought for the retesting in this study.

## Results

### Descriptive analysis

Ct values were generated for 589 IID cases and 159 healthy controls, who were infected with genogroup II noroviruses; 92 of the IID cases were positive by electron microscopy and 497 were negative by electron microscopy but subsequently positive by RT-PCR. IID cases were aged up to 94 years and controls up to 84 years; 40% of IID cases and 60% of controls were aged less than five years.

**Figure 1:**
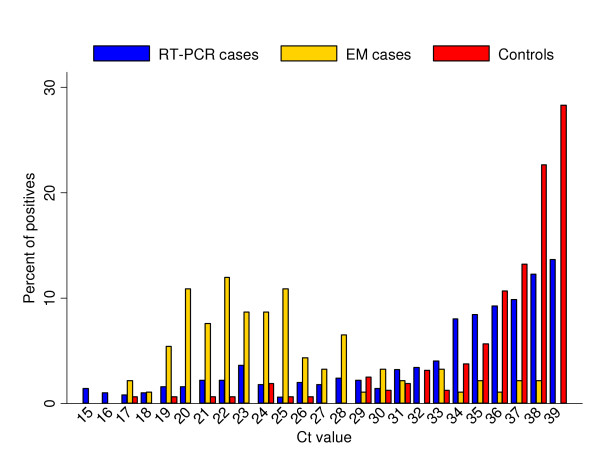
**Percentage distribution of real time RT-PCR Ct values in IID cases and controls**. Low Ct values correspond to high viral loads; the viral load decreases with increasing Ct value. 'EM cases' are IID cases positive by electron microscopy, 'RT-PCR cases' are IID cases negative by electron microscopy and subsequently positive by RT-PCR. Sample sizes: EM cases = 92, RT-PCR cases = 497, controls = 159.

The median Ct value was lower in IID cases (median 34) than in controls (median 38) (Table 2). The difference compared to controls was greatest for IID cases positive by electron microscopy (median 24); there was very little overlap in the distribution of Ct values in electron microscopy positive IID cases and controls (Figure 1). The distribution of Ct values for the IID cases who were negative by electron microscopy and subsequently RT-PCR positive overlaps substantially with the controls, although a small proportion have the higher viral loads seen in the electron microscopy positive IID cases (Figure 1, Table 2).

**Table 2:** Ct values in genogroup II norovirus positive IID cases and healthy controls.

Method of norovirus detection	IID Cases			Controls			Rank-sum test p-value comparing cases to controls
		
	Median Ct value	Ct value IQR	Sample size	Median Ct value	Ct value IQR	Sample size	
All ages							
All	34	25–37	589	38	35–39	159	<0.0001
Electron microscopy	24	21–27.5	92				<0.0001
RT-PCR (Electron microscopy negative)	35	29–38	497				<0.0001
							
< 5 years							
All	34	26–37	253	37	34–38	92	<0.0001
Electron microscopy	23	21–25	48				<0.0001
RT-PCR (Electron microscopy negative)	35	32–37	205				0.0001
							
5 years +							
All	34	25–38	334	38	36–39	67	<0.0001
Electron microscopy	25	22–28.5	44				<0.0001
RT-PCR (Electron microscopy negative)	35	27–38	290				<0.0001

The rank-sum tests for electron microscopy and RT-PCR positive IID cases compare them to all controls. Age was not recorded for two IID cases. IQR is the interquartile range.

### ROC analysis

The numbers of specimens meeting the inclusion criteria for each of the reference groups are shown in Table 3.

**Table 3:** ROC analysis results.

Reference groups used	Optimal Ct cut-off	Youden Index	Sensitivity (95% CI)	Specificity (95% CI)	AUC	Sample size	
						
						Reference positive	Reference negative
Ref positive 1 Ref negative 1							
All	31	0.77	0.88 (0.65–1.00)	0.89 (0.84–0.94)	0.93	92	159

aged <5 years	30	0.80	0.94 (0.84–1.00)	0.86 (0.79–0.93)	0.93	48	92

aged >5 years	33	0.83	0.89 (0.79–0.98)	0.94 (0.88–1.00)	0.96	44	67

Ref positive 2							
Ref negative 1							
	31	0.61	0.72 (0.66–0.79)	0.89 (0.84–0.94)	0.87	169	159
Ref positive 3							
Ref negative 2							
	31	0.29	0.43 (0.39–0.47)	0.86 (0.77–0.94)	0.64	524	64

The reference groups are described in Table 1. AUC is the area under the curve.

**Figure 2:**
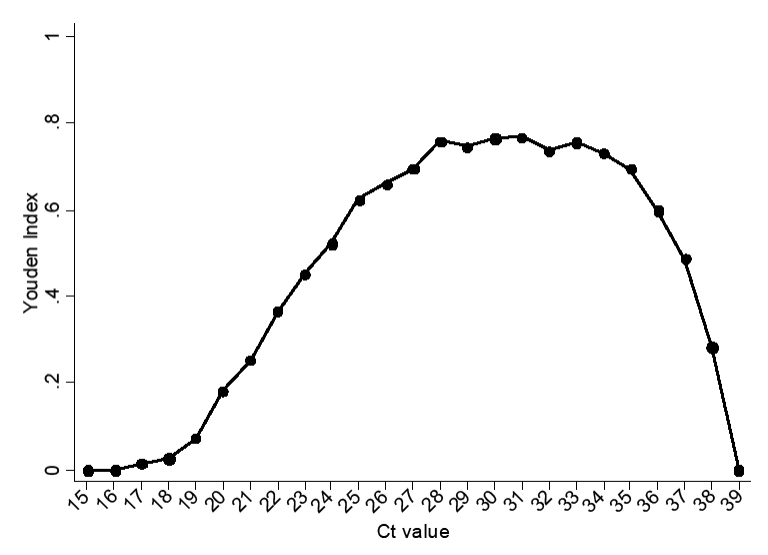
**Youden Index from ROC analysis for reference positive group 1 and reference negative group 1**. Reference positive group 1 were electron microscopy positive IID cases and reference negative group 1 were RT-PCR positive healthy controls.

The optimal cut-off for attributing illness to genogroup II noroviruses in IID cases was at Ct value 31, corresponding to the maximum Youden index for the ROC analysis with reference positive group 1 and reference negative group 1 (Figure 2).

Using this cut-off, IID cases with Ct values of 31 or below are classified as 'positive' for norovirus-associated IID: they have disease caused by norovirus. IID cases with Ct values above 31 are classified as 'negative' for norovirus-associated IID: they have disease but their norovirus infection was not the cause of their symptoms.

The optimal cut-off for children aged less than five years was at Ct value 30, whereas for older children and adults it was at Ct value 33 (Table 3). There was some evidence of a difference in Ct value distribution between electron microscopy positive IID cases in these two age groups (rank sum test p = 0.036), with the median in children aged less than five years at Ct value 23 and at Ct value 25 for older children and adults (Table 2). This indicates that the different cut-offs may reflect a true difference in viral load between these age groups.

The optimal cut-off (all ages) was also at Ct value 31 when RT-PCR positive cases with no other pathogen detected and early specimen collection were included in the reference positive group (reference positive group 2) (Table 3). This was also true for the age-group specific ROC analyses (data not shown). The optimal cut-off was also at Ct value 31 when norovirus positive IID cases who were negative for other commonly tested enteric pathogens were used as the reference positive group (reference positive group 3), and the bacterial culture positive IID cases were used as the reference negative group (reference negative group 2).

**Figure 3:**
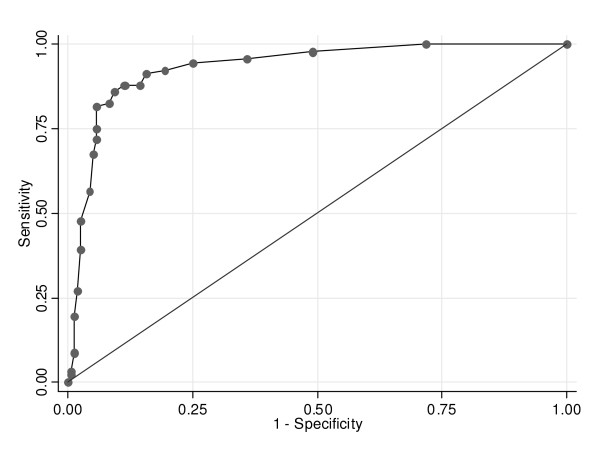
**ROC plot for reference positive group 1 and reference negative group 1**. Reference positive group 1 were electron microscopy positive IID cases and reference negative group 1 were RT-PCR positive healthy controls. The diagonal line represents a ROC plot for a test with no discriminatory power.

The Ct values discriminated well between reference positive group 1 and reference negative group 1, because the area under the ROC curve was close to the maximum value of one (Figure 3, Table 3). The discriminatory power of the Ct values was poorer for the ROC analysis when RT-PCR positive cases with no other pathogen detected and early specimen collection were included in the reference positive group (reference positive group 2). The discriminatory power was very low for distinguishing between reference positive group 3 and reference negative group 2 because the area under the curve was close to 0.5, which is indicative of a test with no discriminatory power.

## Discussion

In this study we have demonstrated a difference in viral load between symptomatic and asymptomatic norovirus infection. A substantial proportion of IID cases who were positive only by RT-PCR had viral loads equivalent to those in healthy controls. This supports the hypothesis that norovirus is not always the cause of illness where it is detected by RT-PCR. We have shown that it is possible to use the viral load in clinical specimens to indicate where norovirus is the most likely cause of illness, by selecting a cut-off for the norovirus real time RT-PCR assay. We have also shown that the method of cut-off selection can be adapted for use with specimens that are routinely received and tested in clinical laboratories, to help other laboratories develop in-house cut-offs for their assays. This is essential because there is substantial variability between UK virology reference laboratories in the Ct values produced from standard reference specimens [31]; the same cut-off may not be appropriate for all laboratories because of these differences in assay performance.

A major strength of this study is the availability of specimens from healthy controls. There are few community studies of IID with large control groups available, but they are essential for interpreting the RT-PCR data in IID cases. Importantly, it has been possible to validate the use of bacterial culture positive IID cases as a reference negative group, by comparison to the ROC analysis using healthy controls; this removes the need to collect further control specimens in future studies. We have also shown that RT-PCR positive IID cases, who are negative for other common bacterial, protozoal and viral pathogens, are a suitable reference positive group, so that the method can be used by laboratories without EM testing facilities. These reference groups can now be used by other laboratories for development of cut-offs for their assays.

The area under the ROC curve for the alternative reference groups is very low, possibly because the viral loads in many of the IID cases in the reference positive group were not representative of symptomatic norovirus infection; this is reflected in the low sensitivity for the cut-off at Ct value 31 when using these groups in the ROC analysis. However, selection of an appropriate cut-off is the main aim of this method and we have shown that this is possible with these reference groups. It is also important that the diagnostic performance (sensitivity, specificity, predictive values) of the cut-off is determined using an independent dataset, which was not possible in this study; the sensitivity and specificity calculated in the ROC analysis may misrepresent the performance of the cut-off in clinical application, because the cut-off is selected by optimising the diagnostic accuracy compared to the gold standard [32,33].

The specimens used in this study were originally collected during the mid 1990s and the viral RNA may have degraded during the prolonged storage and repeated freeze-thaw cycles for re-testing. Therefore the cut-off developed here should not be directly applicable to real-time RT-PCR results from fresh specimens without validation. Similarly, the cut-off should not be applied to assays with different protocols, because the Ct values may not equate to the same viral load per gram of faeces. It is unlikely, however, that there will have been differential degradation of RNA between specimens during storage, so it is still valid to compare the viral load between specimens in this collection, and to assume that the relative differences observed between IID cases and controls are a true reflection of symptomatic and asymptomatic infection. It is also important to note that any cut-off in viral load can only be applied to specimens collected from IID patients during acute symptoms, when the viral load is representative of disease aetiology. After symptoms resolve in norovirus-associated IID, the viral load quickly drops to levels seen in asymptomatic infection [12] and the predictive value of the cut-off will be greatly reduced.

The cut-off developed here is not applicable to two of the rarer genotypes in genogroup II (GII-7 and GII-8), because the real time RT-PCR assay has poorer efficiency (a higher detection limit) for these genotypes (J. Gray, personal communication), so the Ct values do not represent the same faecal viral loads as for the other genotypes. At a population level, the degree of misclassification would be small because of the low prevalence of GII-7 and GII-8 [34-36]. However, correct identification of illness caused by these genotypes may be important for clinical management, but would require development of genotype-specific cut-offs. Similarly, we have excluded genogroup I noroviruses from this analysis because the efficiency of the assay is highly variable for genotypes within in this genogroup. Development of a cut-off for GII-7 and GII-8 or genogroup I noroviruses would require collection of sufficient specimens for genotype-specific ROC analyses; clinical application would require genotyping to be part of routine diagnosis, which may not be economically or logistically feasible. Further work is also needed to characterise the kinetics of the real time RT-PCR assay, to determine whether a Ct value of 31 translates to the same faecal viral load for all genogroup II genotypes with the same assay efficiency. Selection of a single cut-off may also not be appropriate if the Youden index is similar for a range of Ct values between 28 and 33, as was the case in this analysis. With a larger sample size, in future studies, there may be better power to discriminate between potential cut-offs in this range. Nevertheless, the cut-off provides a major improvement in diagnostic specificity compared to the current qualitative use of RT-PCR in norovirus diagnosis.

The causal relationship between disease symptoms and viral load has not been established. However, if the relationship between the occurrence of disease and viral load is consistent, regardless of whether high viral loads are a cause or a consequence of disease, viral load will be a good marker of norovirus-associated IID and the approach developed here is valid. Viral load is routinely used to predict outcome and guide clinical management for a number of viruses that cause chronic infections, such as Epstein-Barr virus [37] and cytomegalovirus [38] in transplant patients, HIV [39], hepatitis C [40] and HTLV [41]. However this is the first time, to our knowledge, that viral load has been used as a tool for diagnosing enteric viruses as the cause of acute IID.

## Conclusion

As PCR diagnosis is applied to an increasing number of viral pathogens, the debate is growing about the clinical interpretation of positive results and the utility of PCR in diagnostic services [10,42,43]. PCR has many advantages over traditional diagnostic methods, including higher throughput, shorter turnaround time, adaptability to new strains and production of data for molecular epidemiological surveillance. It is therefore important to ensure that clinically informative results are produced from PCR assays, to provide a high standard of patient care alongside these other benefits. The method developed here shows that the real-time RT-PCR output for norovirus can be used to attribute disease to norovirus in IID cases, where simple detection may not be sufficient to give a confident diagnosis of norovirus-associated IID. This semi-quantitative approach to diagnosis can improve both the accuracy of community-based estimates of norovirus associated IID incidence and the interpretability of diagnostic results provided to clinicians from clinical virology laboratories. However it is important that clinical and epidemiological information is considered in the diagnosis of disease aetiology for individual patients with Ct values close to the cut-off.

Independent validation of this method is required prior to application in other studies and laboratories; we have provided a method for validation without the need for collection of specimens from healthy controls or further use of EM. The method may also be useful for other viral pathogens, for which the same problems with the interpretability of PCR have been described. Future work will focus on applying this approach for estimation of norovirus associated IID incidence and describing the implications for diagnosis of norovirus outbreaks.

## Abbreviations

AUC: area under the ROC curve; Ct: cycle threshold; ELISA: enzyme linked immunosorbent assay; EM: electron microscopy; IID: infectious intestinal disease; IQR: interquartile range; PCR: polymerase chain reaction; ROC: receiver operating characteristic; RT-PCRL: reverse transcription-polymerase chain reaction.

## Competing interests

The authors declare that they have no competing interests.

## Authors' contributions

GP performed analyses and drafted the manuscript. CT and BL advised on analysis. JG and MIG led the laboratory work and advised on analysis. JG and DB led the study design. All authors contributed to the drafting and revisions of the manuscript.

## Pre-publication history

The pre-publication history for this paper can be accessed here:

http://www.biomedcentral.com/1471-2334/9/63/prepub

## Supplementary Material

Additional File 1 **Testing Summary**. Summary of specimen processing and testing. Click here for file
