# Supplementary material for: Diagnosing norovirus-associated infectious intestinal disease using viral load
Source: BMC Infect Dis. 2009 May 14;9:63. doi: 10.1186/1471-2334-9-63 (PMC2698835; doi:10.1186/1471-2334-9-63)

Additional file 1: Summary of specimen processing and testing

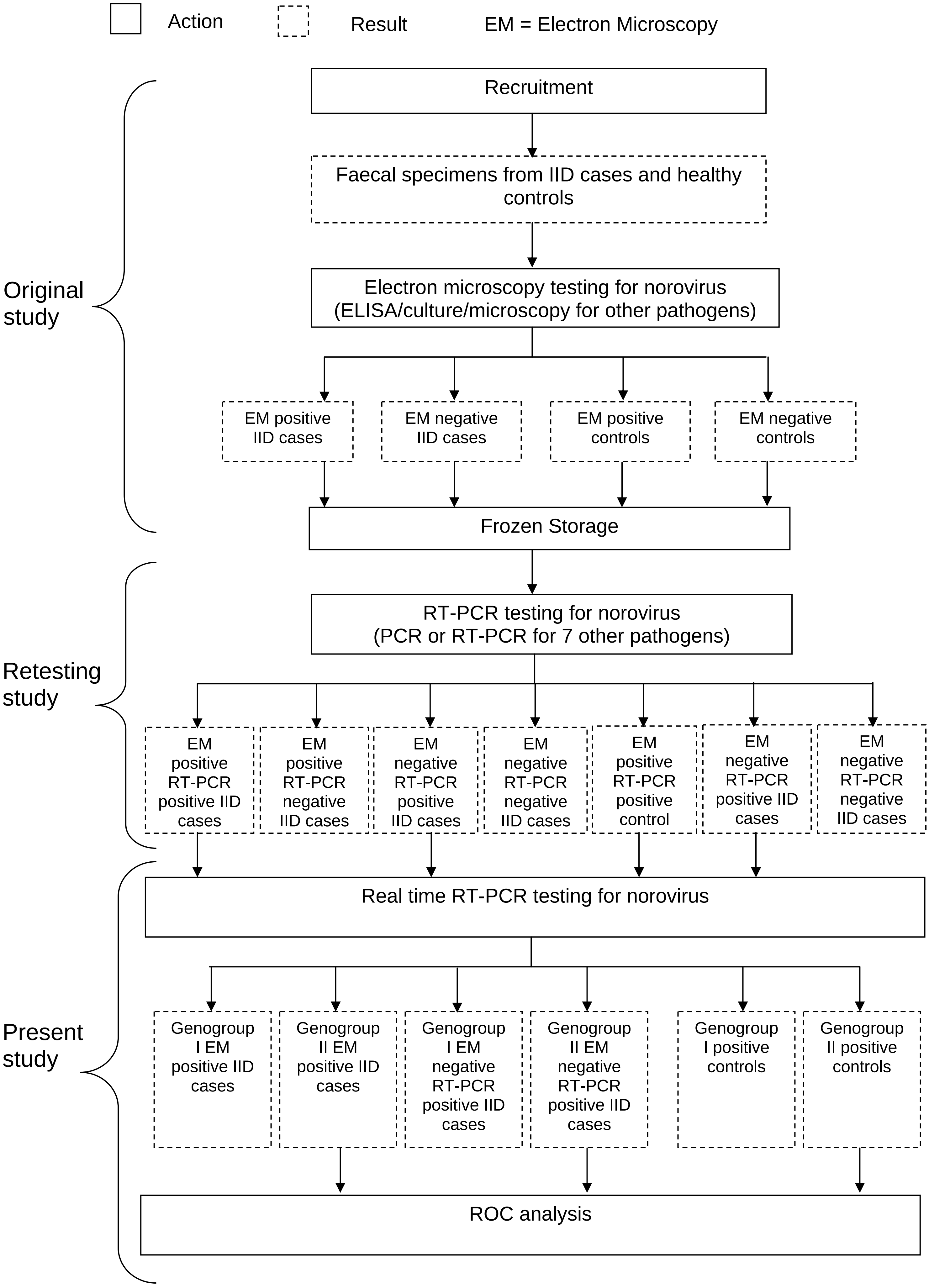

Supplement: Additional File 1 — Testing Summary. Summary of specimen processing and testing. [file 1471-2334-9-63-S1.pdf]
